# An Investigation on the Efficacy of Glucagon-Like Peptide 1 Receptor Agonists Drugs in Reducing Urine Albumin-to-Creatinine Ratio in Patients With Type 2 Diabetes: A Potential Treatment for Diabetic Nephropathy

**DOI:** 10.7759/cureus.36438

**Published:** 2023-03-20

**Authors:** Chetan Yarlagadda, Mohamed Abutineh, Akshay J Reddy, Alec B Landau, Levi M Travis, Cameron G Perrone, Ali Idriss, Rakesh Patel

**Affiliations:** 1 Biomedical Sciences, Florida Atlantic University Charles E. Schmidt College of Medicine, Boca Raton, USA; 2 Medicine, Edward Via College of Osteopathic Medicine, Spartanburg, USA; 3 Medicine, California University of Science and Medicine, Colton, USA; 4 Health Sciences, California Northstate University, Rancho Cordova, USA; 5 Medicine, University of Miami Miller School of Medicine, Miami, USA; 6 College of Science, Florida Atlantic University, Boca Raton, USA; 7 Internal Medicine, East Tennessee State University Quillen College of Medicine, Johnson City, USA

**Keywords:** renoprotection, type 2 diabetes mellitus, uacr, glp1-ra, diabetic nephropathy

## Abstract

As diabetes mellitus becomes increasingly prevalent globally, so does diabetic nephropathy, a complication leading to one of the world’s leading causes of end-stage renal disease (ESRD). Current research has linked an increase in the urine albumin-to-creatinine ratio (UACR), a marker for kidney damage, to a greater risk of adverse renal outcomes and ESRD in patients with diabetes. Of the diabetes medications studied and implemented in clinical settings, glucagon-like peptide-1 receptor agonist (GLP1-RA) drugs have been shown to not only help control HbA1c in diabetes but have also demonstrated numerous cardiovascular, hepatic, and renal benefits. The objective of our study was to assess the efficacy of GLP1-RA drugs in reducing UACR in patients with type 2 diabetes mellitus (T2 DM) to determine if GLP1-RAs could be used to provide renoprotection in diabetic nephropathy in addition to their glucose-lowering effects. Upon a comprehensive review of the literature, we conducted a statistical analysis to determine the efficacy of GLP1-RA monotherapy and combination therapy in reducing UACR in comparison to placebo and insulin glargine. Of the studies analyzed, GLP1-RAs exhibited a statistically significant effect in reducing UACR in comparison to a placebo but not in comparison to insulin glargine. GLP1-RA combination therapy (GLP1-RA used with either insulin glargine, metformin, or dapagliflozin) did not exhibit statistically significant UACR reductions in comparison with insulin glargine. However, GLP1-RA combination therapy showed a trend suggestive of being more effective than insulin glargine in reducing UACR, but due to the limited literature studying this treatment method, further studies in a more focused group of patients with diabetic nephropathy may produce stronger and more definitive results. GLP1-RA monotherapy or combination therapy has been determined to be an effective method for reducing UACR and decreasing the incidence of adverse renal outcomes associated with diabetic kidney disease. GLP1-RA therapy could serve as an alternative treatment in diabetic nephropathy to insulin glargine, which carries a higher risk of hypoglycemia and unintentional weight gain while potentially being less cost-effective.

## Introduction and background

Diabetes and chronic kidney disease

Diabetes mellitus (DM) is a chronic metabolic disorder characterized by elevated blood glucose (hyperglycemia). With DM afflicting approximately 11% of the global population and the percentage expected to grow rapidly over the coming years, there has never been a stronger need to focus on diabetes and its multitude of adverse health effects [1]. The majority of cases of diabetes fall into two etiopathogenetic categories [2]. Type 1 diabetes (T1 DM) is caused by an absolute deficiency of insulin secretion owing to an autoimmune pathologic process occurring in the pancreatic islets. Type 2 diabetes mellitus (T2 DM) is due to both an increased resistance to insulin as well as insufficient compensatory insulin secretion. Other causes of diabetes may include gestational diabetes mellitus, genetic disorders, and those caused by drugs, chemicals, or infection [1]. DM can cause many associated comorbidities, including adverse cardiovascular, gastrointestinal, and renal outcomes. Cardiovascular complications of DM include an increased risk of heart disease and stroke, while gastrointestinal complications include gastroparesis, which is delayed emptying of the stomach, and diarrhea [3]. Diabetic nephropathy develops in approximately 40% of those with diabetes, making it a leading cause of end-stage renal disease (ESRD) globally [4]. There are two key markers for chronic kidney disease (CKD): the urine albumin-to-creatinine ratio (UACR) and the estimated glomerular filtration rate (eGFR). UACR estimates 24-hour urine albumin excretion. UACR values above 30 mg/g are positive for albuminuria and are sensitive markers for CKD [5]. Drugs that decrease UACR are associated with improved cardiovascular outcomes in addition to improved renal outcomes [5]. There are several treatments currently available for diabetes, including sodium-glucose cotransporter-2 (SGLT2) inhibitors, dipeptidyl peptidase-4 (DPP-4) inhibitors, biguanides, thiazolidinediones (TZDs), and glucagon-like peptide-1 receptor agonists (GLP-1RAs). Treatment options for diabetic patients with ESRD are limited: for patients with deteriorated renal function, common oral hypoglycemic agents like metformin are not recommended [6]. These treatments have been shown to have varying effects on kidney disease in diabetics. For example, SGLT2 inhibitors and TZDs have been shown to substantially reduce albuminuria, although TZDs have uncertain effects on patients with heart failure, angina, myocardial infarction, and general cardiovascular mortality [7-8]. GLP1-RAs are a class of incretin-based therapies that have been widely studied for their effects on HbA1c, cardiovascular disease (CVD), and nonalcoholic fatty liver disease (NAFLD). GLP-1RAs have a glucose-dependent mechanism of action that stimulates pancreatic β cells, delays gastric emptying, and suppresses glucagon release [9]. Emerging studies have found that exenatide can decrease urinary albumin excretion in patients with type 2 diabetes [10]. Insulin therapy has for many years remained the gold standard in controlling diabetes, but it has also proven effective in reducing urine albumin excretion and does not negatively hinder kidney function as it is cleared by the kidneys [11]. However, the extent to which GLP-1RAs are able to lower UACR to potentially prevent the incidence of ESRD or the worsening of diabetic kidney disease (DKD) has not been fully elucidated.

Renoprotective mechanisms of GLP1-RAs

One of the mechanisms by which GLP-1RAs have been shown to have renal-protective effects is natriuresis. Natriuresis is the increased urinary excretion of sodium, which can lead to a reduction in blood pressure and a decrease in the workload on the heart. GLP-1RAs phosphorylate and inactivate the Na+/H+ exchanger 3 in the proximal tubule of the nephron, decreasing Na+ reabsorption and causing natriuresis [12]. Further, cardiac GLP-1R activation by liraglutide has been shown to promote the secretion of atrial natriuretic peptide (ANP) [13]. Oxidative stress in diabetic nephropathy is in part mediated by protein kinase C (PKC) activation and protein kinase A (PKA) inhibition [14]. In addition to natriuresis, GLP-1RAs have been shown to protect glomeruli and tubules by inhibiting PKC and activating PKA [15]. The activation of PKA inhibits nicotinamide adenine dinucleotide phosphate and produces cyclic adenosine monophosphate [15]. Second, GLP-1RAs stimulate the release of anti-inflammatory cytokines such as tumor necrosis factor-alpha (TNF-a) and interleukin-6 [16]. Specifically, liraglutide inhibits TNF-a mediated nuclear factor kappa B activation in podocytes [17]. Through an indirect mechanism, the increased levels of ANP due to GLP-1RA therapy also increase the expression of antioxidant enzymes such as superoxide dismutase and glutathione peroxidase [18]. In general, the renoprotective effects of GLP-1RAs are heavily based on the prevention of oxidative stress and inflammation. Renal fibrosis is a common route of CKD progression to ESRD. Exendin-4, a GLP-1RA commonly known as Exenatide, has been found to ameliorate renal fibrosis by inhibiting miR-192, a microRNA [19]. Liraglutide has been shown to suppress transforming growth factor-beta 1 and its downstream signaling pathways, reducing tubulointerstitial fibrosis [20]. These protective effects against renal fibrosis are related to diminishing the epithelial to mesenchymal transition of tubular cells [20-21]. However, the effects of GLP-1RAs on UACR need to be studied further. Currently, there is limited information available on the exact mechanisms by which GLP-1RAs reduce albuminuria, and more research is needed to fully elucidate the potential benefits of these medications in the treatment of renal impairment in patients with DM. Nevertheless, current literature suggests that GLP-1RAs have a promising potential as a renal-protective agent in the treatment of this patient population.

## Review

Methods

**Figure 1:**
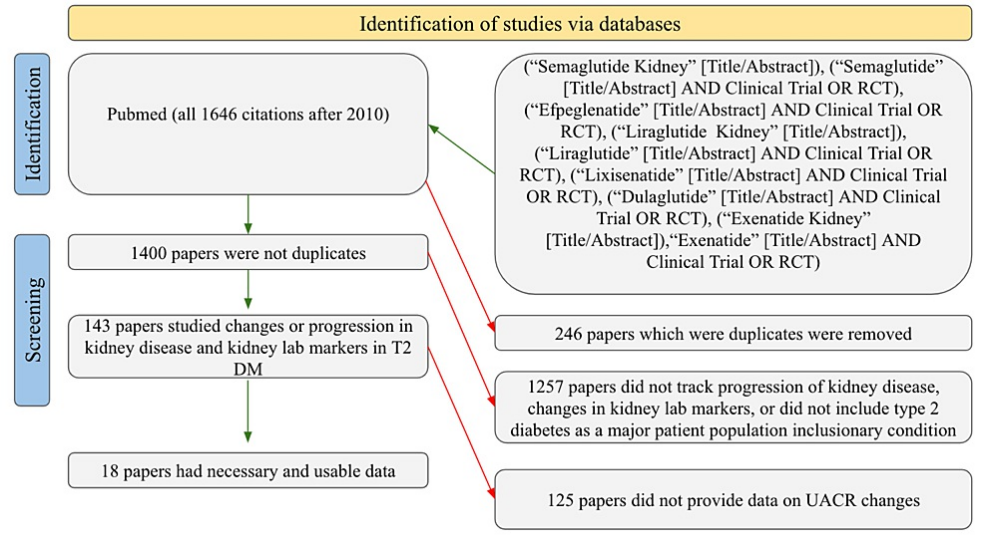
PRISMA diagram to provide a visual representation of the study screening process PRISMA: Preferred Reporting Items for Systematic Reviews and Meta-Analyses; RCT: randomized clinical trial. Figure independently designed by authors.

A comprehensive search was conducted on PubMed to obtain an all-encompassing query on using various GLP1-RAs in patients with T2 DM and their respective effects on changes in UACR. In the identification stage, using a combined total of nine different searches, an aggregate total of 1646 papers published after 2010 were identified. Citations were imported to Mendeley Reference Manager, through which duplicate studies were manually found and removed, resulting in a reduction of 246 papers and an aggregate total of 1400 unique papers. Of the non-duplicate papers, each title and abstract were individually scanned for relevance, and it was found that only 143 papers studied relevant topics. One hundred and twenty-five papers were further eliminated due to specific calculations in the UACR not being provided, so 18 papers were left to be used for analysis. Figure 1 illustrates the compilation of each step of the aforementioned screening process. Of these 18 papers, information was summatively collected regarding the specific GLP1-RA used, dosage, frequency of administration, method of administration, comparator compound to the GLP1-RA, major inclusionary conditions for the patient population in the study, total patient population size, length of follow-up, and changes in UACR in both the GLP and comparator groups. The papers included in this study were either clinical trials or retrospective observational studies. Clinical trials were used to understand the effects of GLP1-RAs compared to a placebo or a current medication whose effects are understood in DKD. Retrospective observational studies were used to understand the real-world clinical application of GLP1-RAs and their efficacy in a non-standardized medical setting. Graph creation and statistical analyses were conducted using GraphPad Prism 9 software. Statistical analyses included a t-test and the removal of outliers identified by GraphPad Prism 9. Two GLP1-RA values were removed from the statistical analysis as they were identified as outliers. The two retrospective observational studies without a comparator were not included in the statistical analysis, as the lack of a comparator for GLP1-RAs does not allow for a standardized comparison of the change in baseline UACR values.

GLP1-RAs and insulin therapy in diabetic nephropathy

From 2013 to 2016, in the US alone, an estimated 37% of adult diabetics presented with CKD [4]. Over half of those patients were in stages 3-4, categorized as having moderate-to-severe CKD [4]. With the rapid growth in diabetes and the alarming risk of DKD, past guidelines and medications must be revisited and improved upon. Current research on DKD suggests that the greater the percentage reduction in UACR, the greater the reduction in the risk of kidney failure [5]. More notably, the greater the UACR is during the diagnosis of DKD, the greater the risk of an eventual adverse renal event [5]. Recent advisory clinical guidelines have slowly shifted to recommend early prescriptions of GLP1-RAs for DKD [22]. A likely explanation of the change in clinical guidelines, which suggests an early prescription in people with diabetes at risk of DKD, is the associated drop in UACR with the use of GLP1-RAs, thereby lowering the risk of kidney failure and reducing the risk of adverse renal events.

Insulin therapy is the current gold standard of glycemic control in patients with uncontrolled diabetes and is indicated to begin if maximally dosed dual oral therapy is unsuccessful [23]. It remains a cornerstone medication in glycemic control, especially in DKD, for its albuminuria-reducing effects [11]. However, it is essential to note that insulin therapy is not suitable for all patients, such as those with non-insulin-dependent DM, for whom insulin therapy is a last resort [24]. Cases such as these bring forth the need for new treatments that improve upon insulin therapy alone in treating DKD and reducing UACR.

While current research does not have a definitive stance on the effect of GLP1-RAs on GFR, there has been strong evidence to show that they provide a marked reduction in UACR, notably seen by 14 of the 16 unique trials and observational studies analyzed in Table 1 [25-42]. In Table 1, four papers used insulin glargine rather than placebo as a control to compare against the efficacy of the GLP1-RA being studied [36,38-39,41]. Presumably, insulin glargine was commonly used as a comparator drug to GLP1-RAs since insulin has been shown to reduce albuminuria along with its primary ability to reduce blood glucose and could act as a positive control to which the efficacy of GLP1-RAs could be compared to [11]. GLP1-RA treatment compared to placebo, the negative control, could demonstrate an ability to decrease UACR si, but GLP1-RA treatment compared to insulin therapy, the positive control, could demonstrate superiority in the treatment of DKD compared to current staples in the treatment of glycemic control and DKD.

**Table 1:** Outcomes of UACR changes in clinical trials and observational studies using GLP1-RAs CHF: congestive heart failure, mg: milligram, N/A: not applicable, SC: subcutaneous, UAER: urinary albumin excretion ratio, w/: with

TRIAL, Author (Year)	GLP1- RA Drug Studied	GLP1-RA Dosage, Method, and Frequency of Administration	Comparator	Major Inclusionary Conditions	Sample Size	Length of Follow up	Urinary Albumin-Creatinine Ratio (UACR) Changes
PIONEER 5, Mosenzon et al. [25]	Semaglutide	14 mg, oral, daily	Placebo	T2 DM, eGFR 30-59 mL/min per 1.73 m^2^	324	26 weeks	14% decrease w/ Semaglutide. 19% Increase w/ Placebo.
Bueno et al. [26]	Semaglutide	1 mg, SC, weekly	N/A	T2 DM, CKD w/ eGFR > 15 mL/min per 1.73 m^2^	122	52 weeks	53% decrease w/ Semaglutide.
SUSTAIN 6, Marso et al. [27] and Mann et al. [28]	Semaglutide	(1) 0.5 mg, SC, weekly; (2) 1 mg, SC, weekly	Placebo	T2 DM w/ CVD or or CHF or CKD stage 3+	3297	109 weeks	2.7% decrease w/ Semaglutide 0.5 mg, 14.2% decrease w/ Semaglutide 1 mg. 30.2% increase w/ placebo.
AMPLITUDE-O, Gerstein et al. [29]	Efpeglenatide	4 mg, SC, weekly	Placebo	T2 DM w/ CVD or middle-age w/ CKD	4076	94.4 weeks	21% decrease w/ Efpeglenatide.
ELIXA, Pfeffer et al. [30]	Lixisenatide,	20 µg, SC, daily	Placebo	T2 DM w/ acute coronary syndrome	6068	108 weeks	24% increase with Lixisenatide, 34% increase w/ placebo.
LEADER, Marso et al. [31] and Persson et al. [32]	Liraglutide	1.8 mg, SC, daily	Placebo	T2 DM w/ CVD history or CVD risk factors	9340	198.3 weeks	15% reduction w/ Liraglutide, 10% increase in placebo. Results were after 1 year of follow up.
LIRA-RENAL, Davies et al. [33]	Liraglutide	1.8 mg, SC, daily	Placebo	T2 DM w/ moderate renal impairment	279	26 weeks	13% decrease w/ Liraglutide, 5% increase w/ placebo.
SCALE, Davies et al. [34]	Liraglutide	(1) 3.0 mg, SC, daily; (2) 1.8 mg, SC, daily	Placebo	T2 DM	827	68 weeks	18.4% decrease w/ Liraglutide 3 mg, 10.8% decrease w/ Liraglutide 1.8 mg, 2.3% decrease with placebo.
de Lucas et al. [35]	Liraglutide	N/A	N/A	DM2, CKD stage 3	23	52 weeks	36.7% decrease w/ Liraglutide.
AWARD 7, Tuttle et al. [36]	Dulaglutide	(1) 1.5 mg, SC, weekly; (2) 0.75 mg, SC, weekly	Insulin Glargine	T2 DM w/ CKD Stage 3-4	576	56 weeks	20.1% decrease w/ Dulaglutide 1.5 mg, 13% decrease w/ Dulaglutide 0.75 mg, 22.5% decrease with Insulin
REWIND, Gerstein et al. [37] and Persson et al. [32]	Dulaglutide	1.5 mg, SC, weekly	Placebo	T2 DM w/ CVD history or CVD risk factors	9901	281.8 weeks	18% reduction w/ Dulaglutide
DURATION 3, Diamant et al. [38]	Exenatide	2 mg, SC, weekly	Insulin Glargine	T2 DM	456	26 weeks	15.4% decrease w/ Exenatide, 13% decrease w/ Insulin.
Wang et al. [39]	Exenatide w/ Insulin Glargine	10 µg, SC, twice daily	Insulin Glargine	T2 DM w/ eGFR>30 and UAER >0.3 g/24h	92	24 weeks	80.9% decrease w/ Exenatide plus Insulin, 52.5% decrease w/ Insulin only.
Zhang et al. [40]	Exenatide w/ Metformin	10 µg, SC, twice daily	Glimepiride w/ metformin	T2 DM	31	16 weeks	38% decrease w/ Exenatide, no reported change w/ Glimepiride.
Pawaskar et al. [41]	Exenatide	SC, twice daily	Insulin Glargine	T2 DM	5366	52 weeks	306% increase w/ Exenatide, 26.3% decrease w/ Insulin.
DECREASE, van Ruiten et al. [42]	Exenatide only or exantide w/Dapagliflozin	10 µg, SC, twice daily (used for both exenatide only and exantide w/ Dapagliflozin groups)	Dapagliflozin or placebo	T2 DM	66	16 weeks	39.6% decrease w/ Exenatide and Dapagliflozin, 18.1% decrease w/ Dapagliflozin only, 15.6% decrease w/ Exenatide only, and 11% decrease w/ placebo.

UACR reduction in GLP1-RA monotherapy and insulin therapy

Upon statistical analysis, as seen in Table 2, it was determined that the use of a GLP1-RA drug was able to significantly decrease UACR values compared to a placebo in T2 DM patient populations. In Table 1, three studies using a different GLP1-RA documented the effect of varying drug doses within the trial [27,34,36]. Of the aforementioned studies, each of the individual GLP1-RA drugs exhibited a greater reduction in UACR as the dosage increased, suggesting a class-wide dose-dependent effect not limited to one GLP1-RA drug [27,34,36]. Unfortunately, adverse symptoms such as nausea and vomiting can occur with maximal doses of GLP1-RA, and some patients may be unable to tolerate such doses. However, many of the common negative symptoms have been shown to be attenuated with prolonged usage of GLP1-RAs [43]. While the maximally administered dosage appears most beneficial, even doses as low as half the maximally administered dosage prompted considerable decreases in UACR compared to placebo [27,34,36]. In such patients, it stands to reason that even with a lower dosage that is maximally tolerable for a GLP1-RA, they could still receive considerable benefits in reducing UACR. Using anti-emetic drugs, such as metoclopramide and ondansetron hydrochloride, in conjunction with a GLP1-RA reduces symptoms of nausea and vomiting and could aid in adapting to the initial usage of the GLP1-RA, allowing for more patients to be able to escalate their dosage [44]. Further research must be conducted on the acute usage of anti-emetics alongside GLP1-RAs.

**Table 2:** Statistical analysis of the target group means

Group comparison	Mean UACR Change Per Group	T-Value	P-Value
GLP1-RA vs placebo	−18.9% (GLP1-RA) vs 12.1% (placebo)	3.424	0.038
GLP1-RA vs insulin glargine	−18.9% (GLP1-RA) vs -28.6% (insulin glargine)	1.913	0.0799
GLP1-RA combined treatment vs insulin glargine	−52.8% (GLP1-RA combination treatment) vs -28.6% (insulin glargine)	1.59	0.1727

Interestingly, when insulin glargine was used as a comparator drug to GLP1-RAs, there was no statistical difference in the reduction of UACR between the two medications, as seen in Table 2. Though there is no statistical significance between the two, GLP1-RAs carry a significantly lower risk of hypoglycemia and have been shown to induce weight loss, while insulin possesses a higher risk of hypoglycemia, often causing unintentional weight gain [45-47]. Studies have also shown GLP1-RAs to be potentially cost-effective compared to insulin therapy; however, many highly variable factors can determine the cost-effectiveness of insulin and GLP1-RAs [48]. While current guidelines in the use of insulin are effective in glycemic control and the reduction of UACR, GLP1-RA treatment could be a strong alternative in DKD due to their similar efficacy to insulin in reducing UACR with lower financial burdens and health risks.

Efficacy of GLP1 combination therapy

**Figure 2:**
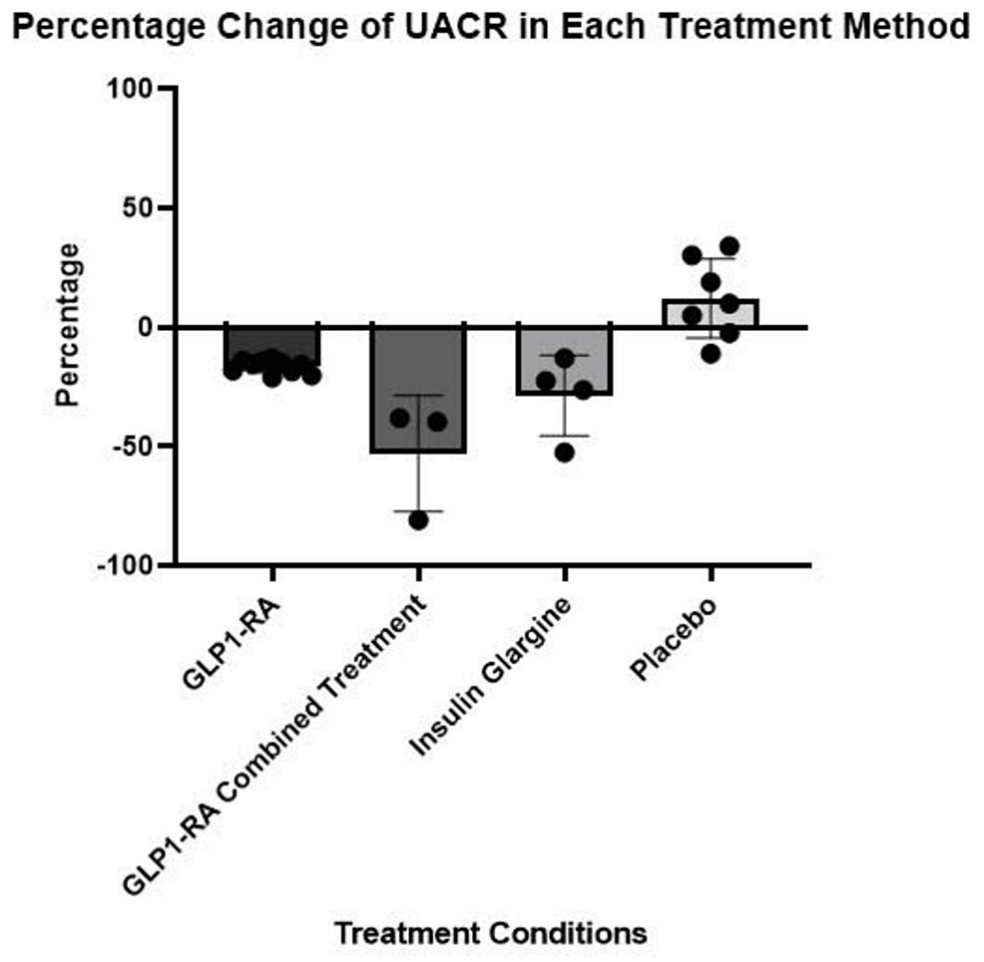
Comparison of mean UACR reductions per treatment condition

The GLP1-RA combined treatment group consisted of three studies from Table 1 that studied exenatide taken in combination with either insulin glargine, metformin (biguanide), or dapagliflozin (SGLT2 inhibitor) [39-40,42]. Literature suggests that GLP1-RAs combined with an antidiabetic drug in a different class, such as an SGLT2 inhibitor, may exhibit a synergistic improvement in glycemic control and provide multi-organ system benefits such as greater improvement of DKD than with GLP1-RA monotherapy [49]. Evaluation of Table 2 revealed that there is no statistical difference between GLP1-RA combination therapy and insulin glargine in reducing UACR. However, Figure 2 depicts a potential trend toward a greater reduction in UACR with GLP1-RA combination therapy, and Table 2 reveals a greater mean UACR decrease of 24.2% versus insulin glargine. Among the GLP1-RA combination studies, there is considerable variance in results, but exenatide used concurrently with insulin glargine induced the single largest reduction in UACR seen in all of the studies in Table 1 [39]. Current research supports the use of GLP1-RA in combination with a lower dose of insulin to more effectively reduce HbA1c while mitigating weight gain from insulin and lowering the risk of hypoglycemia, all with no change in adverse gastrointestinal issues from the use of GLP1-RA independently [50]. Literature on the combined usage of GLP1-RAs and insulin in DKD is limited, but their concurrent use in diabetes is promising and could significantly reduce UACR. Moreover, the two combination therapy studies within Table 1 studying exenatide with metformin and exenatide with dapagliflozin demonstrate these combinations as effective options for reducing UACR. However, they are not as effective as exenatide with insulin and are not statistically greater than insulin alone [40,42].

Current limitations and future applications

Our review provides context to clinicians regarding the use of GLP1-RA drugs in patients with or at risk of DKD to reduce UACR and help prevent adverse renal outcomes or the precipitation of ESRD in patients with T2 DM. Renoprotective mechanisms of GLP1-RAs are not fully understood yet and present a challenge in developing studies that allow for combination therapies with drugs that can provide renoprotection in DKD without increasing the risk of adverse health outcomes. In our review, only three studies used a GLP1-RA medication in combination with another drug for diabetes: an SGLT2 inhibitor, biguanide, or insulin therapy [39-40,42]. In future reviews, authors should aim to provide multiple studies focused on reducing UACR using GLP1-RAs in combination with each varying class of anti-diabetic drugs such as biguanides, sulfonylureas, SGLT2 inhibitors, TZDs, or DPP-4 inhibitors. Another limitation is that two of the papers in our review did not use a comparator compound to evaluate the efficacy of the GLP1-RA being studied. Ultimately, the data were not used to prevent biasing or skewing the statistical analysis and results [26,35]. Despite lacking a comparator, these papers were retrospective observational studies documenting UACR reductions in a clinical setting using GLP1-RAs. Both studies presented a significant reduction, suggesting the strong efficacy of GLP1-RA in clinical settings. Although the small sample size of studies was a notable limitation, a greater problem was the lack of substantive studies focused specifically on patients with DKD. While GLP1-RAs have been extensively studied as a means to reduce HbA1c, their cardiometabolic and hepatic benefits have only been discovered recently, and studies are currently being conducted specifically on the use of GLP1-RAs on DKD. Further investigations should look closely at the ongoing clinical trial FLOW, expected to be completed in August 2024, investigating semaglutide versus placebo in patients with T2 DM and CKD [51]. This study could provide further definitive evidence of the ability of GLP1-RAs to reduce UACR and prevent adverse renal outcomes in DKD patient populations. Future reviews should be conducted with trials studying the use of GLP1-RAs in a focused population of patients with DKD, like the aforementioned FLOW trial, to understand their direct reduction of UACR, adverse renal outcomes, and ESRD incidence.

## Conclusions

This literature review analyzed current studies regarding the use of GLP1-RA drugs and their respective reductions in UACR. The authors completed a review of 18 individual papers that consisted of 16 unique studies on GLP1-RAs and their effectiveness in reducing UACR in T2 DM as a possible tool to treat patients with DKD or at high risk of DKD. While the data showed no significance between the efficacy of GLP1-RA compared to insulin glargine or GLP1-RA combination therapy compared to insulin glargine, the use of GLP1-RAs was statistically more effective than a placebo in reducing UACR. Although there was not a statistical difference in the efficacy of GLP1-RA monotherapy or combination therapy in reducing UACR versus insulin glargine, GLP1-RA treatment is a promising alternative to insulin therapy in DKD due to the lower risk of hypoglycemia, the induced weight loss instead of weight gain with insulin therapy, and a possible reduction in cost. Subsequent reviews should assess the effectiveness of various antidiabetic drugs used concurrently with GLP1-RAs. If further data reveal that GLP1-RA combination therapy is more effective in reducing UACR than GLP1-RA monotherapy or insulin therapy, GLP1-RA combination therapy could be the first line of glycemic control and renoprotection in diabetic patients at risk of progression to CKD. This investigation could inform clinicians of the current capabilities of GLP1-RAs to reduce UACR in diabetic patients, allowing for a reduction in the risk of adverse renal events and ESRD in a population especially prone to such negative outcomes.
